# The Potential of SHAP and Machine Learning for Personalized Explanations of Influencing Factors in Myopic Treatment for Children

**DOI:** 10.3390/medicina61010016

**Published:** 2024-12-26

**Authors:** Jun-Wei Chen, Hsin-An Chen, Tzu-Chi Liu, Tzu-En Wu, Chi-Jie Lu

**Affiliations:** 1 School of Medicine, Chang Gung University, Taoyuan 333, Taiwan; 2 Graduate Institute of Business Administration, Fu Jen Catholic University, New Taipei City 242, Taiwan; 3 School of Medicine, Fu Jen Catholic University, New Taipei City 242, Taiwan; 4 Department of Ophthalmology, Shin Kong Wu Ho-Su Memorial Hospital, Taipei 111, Taiwan; 5 Artificial Intelligence Development Center, Fu Jen Catholic University, New Taipei City 242, Taiwan; 6 Department of Information Management, Fu Jen Catholic University, New Taipei City 242, Taiwan

**Keywords:** myopia, atropine, intraocular pressure, machine learning, SHAP value

## Abstract

*Background and Objectives:* The rising prevalence of myopia is a significant global health concern. Atropine eye drops are commonly used to slow myopia progression in children, but their long-term use raises concern about intraocular pressure (IOP). This study uses SHapley Additive exPlanations (SHAP) to improve the interpretability of machine learning (ML) model predicting end IOP, offering clinicians explainable insights for personalized patient management. *Materials and Methods:* This retrospective study analyzed data from 1191 individual eyes of 639 boys and 552 girls with myopia treated with atropine. The average age of the whole group was 10.6 ± 2.5 years old. The refractive error of spherical equivalent (SE) in myopia degree was base SE at 2.63D and end SE at 3.12D. Data were collected from clinical records, including demographic information, IOP measurements, and atropine treatment details. The patients were divided into two subgroups based on a baseline IOP of 14 mmHg. ML models, including Lasso, CART, XGB, and RF, were developed to predict the end IOP value. Then, the best-performing model was further interpreted using SHAP values. The SHAP module created a personalized and dynamic graphic to illustrate how various factors (e.g., age, sex, cumulative duration, and dosage of atropine treatment) affect the end IOP. *Results*: RF showed the best performance, with superior error metrics in both subgroups. The interpretation of RF with SHAP revealed that age and the recruitment duration of atropine consistently influenced IOP across subgroups, while other variables had varying effects. SHAP values also offer insights, helping clinicians understand how different factors contribute to predicted IOP value in individual children. *Conclusions:* SHAP provides an alternative approach to understand the factors affecting IOP in children with myopia treated with atropine. Its enhanced interpretability helps clinicians make informed decisions, improving the safety and efficacy of myopia management. This study demonstrates the potential of combining SHAP with ML models for personalized care in ophthalmology.

## 1. Introduction

Myopia severity has increased globally, becoming a major concern for ophthalmologists. Currently, it affects 1.4 billion people (22.9% of the population) and is expected to affect 4.8 billion people (49.8%) by 2050 [1]. Studies link myopia incidence to race and other risk factors, with rates rising in both Western and Asian countries [2,3], especially among younger Asian women, according to some studies [4].

Clinicians are focused on controlling myopia progression, with three main treatments for adolescents, namely atropine eye drops, orthokeratology (OK) lenses, and peripheral defocus spectacle lenses [5,6]. These interventions aim to decelerate myopia progression and reduce the risk of associated complications, such as cataracts, retinal detachment, optic atrophy, glaucoma, retinal detachment, chorioretinal atrophy, and lacquer cracks. These conditions can lead to irreversible visual impairment and severe sequelae, leading to blindness.

Atropine effectively controls myopia progression and prevents high myopia [7,8]. However, concerns about the side effects of long-term usage have grown among parents and clinicians [9]. Atropine, a non-selective antimuscarinic agent with high affinity for M1–M5 receptors, causes mydriasis and cycloplegia in the pupillary sphincter ciliary muscle. Common reported side effects of topical atropine include light sensitivity, blurred near vision due to pupil dilation, and temporary accommodation [10,11]. Additionally, atropine may increase resistance through the trabecular meshwork, potentially impeding aqueous humor flow into the Schlemm’s canal [12]. Pupillary dilation has been identified as a predisposing factor for glaucoma [13]. Atropine and other anticholinergic agents may elevate intraocular pressure (IOP), making them contraindicated in patients with glaucoma [14]. In normal children, IOP gradually increases with age, stabilizing at adult levels around age 12 [15]. The safety of this treatment remains a subject of ongoing debate, particularly regarding its potential to elevate intraocular pressure (IOP). It is well established that individuals with myopia are at a higher risk of developing glaucoma, including normotensive glaucoma. Consequently, there is significant clinical interest in establishing reliable prognostic factors to identify contraindications or limitations for atropine use in patients at risk of increased IOP.

Previous studies employing biostatistical methods have conducted retrospective or prospective studies on children with myopia treated with atropine, indicating that atropine treatment does not pose a high risk of IOP exceeding safety thresholds that could lead to glaucoma [16]. Furthermore, machine learning (ML) analyzed the data by extreme gradient boosting (XGB) and found that base IOP was the most influential factor affecting the final IOP [17]. Moreover, our multivariate adaptive regression splines (MARS) study showed that there is a positive correlation in the final IOP when the patients’ base IOP is >14 mmHg [18]. Therefore, in this study, we categorized patients into two subgroups based on a base IOP cutoff of 14 mmHg. This article is based on a previous study [18], which identified the key factors influencing end intraocular pressure (IOP) in children receiving atropine treatment. The prior study highlighted the following variables in order of their impact on end IOP: baseline IOP (base IOP), recruitment duration, age, and previous treatment duration, with base IOP being the most significant factor. Additionally, it suggested that atropine may elevate end IOP in children with a baseline IOP greater than 14 mmHg. We incorporated the more prominent influencing factors from the previous analysis as variables (X1–X8) for further examination of their effects on IOP in myopic children undergoing atropine treatment.

We employed ML and SHapley Additive exPlanations (SHAP) to analyze confounders within these IOP subgroups and evaluate their impact on predicting final IOP. This study, to the best of our knowledge, is the first attempt to implement the SHAP module in adolescent patients with myopia treated with atropine. The SHAP module created a personalized and dynamic graphic to illustrate how various factors (e.g., age, sex, cumulative duration, and dosage of atropine treatment) affect the end IOP. This study aimed to predict the end IOP in myopic patients treated with atropine, providing physicians with more individualized insights to enhance patient management and medication safety.

## 2. Materials and Methods

### 2.1. Study Design and Protocol

**Figure 1:**
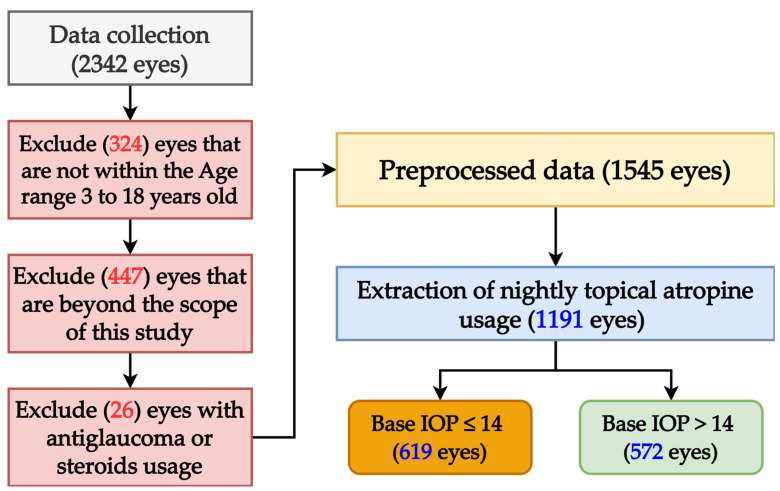
Data preprocessing workflow.

This study, conducted at Shin-Kong Wu Ho-Su Memorial Hospital in Taipei, Taiwan, analyzed data from 2342 eyes of 1171 children diagnosed with myopia and a refractive error of spherical equivalent (SE) less than −10.0D. Data were collected from 1 January 2008 to 31 December 2008, in accordance with the Declaration of Helsinki and approved by the hospital’s Institutional Review Board (IRB 20220706R). Exclusions included 324 eyes from participants aged <3 or >18 years; 447 eyes due to loss of follow-up, use of cycloplegics without atropine or presence of ocular diseases including corneal opacity, traumatic injury, uveitis, congenital cataract, congenital glaucoma, optic nerve atrophy, ocular tumor, or prior surgery; and 26 eyes due to use of steroids or anti-glaucoma medications. After exclusions, 1545 eyes from myopic children with regular nightly atropine use remained; 354 eyes were further excluded for irregular drug usage, leaving 1191 individual eyes from 639 boys and 552 girls for analysis. The average age of the whole group was 10.6 ± 2.5 years old. The refractive error of spherical equivalent (SE) in myopia degree was base SE at 2.63D and end SE at 3.12D. The change in myopia degree was −0.48D. Since this article is an extended study based on the order of variables in the previous article, the most important factor affecting end IOP by atropine is base IOP [17]. A previous study suggested that atropine may increase the end IOP in children with a base IOP greater than 14 mmHg [18]. Therefore, base IOP 14 mmHg was used as the basis for grouping, and then the other significant influencing factors in the previous article were further defined as variables (X1–X8) affecting IOP in myopic children undergoing atropine treatment in this article [17,18]. As for the initial myopic degree, since the importance of base SE was less obvious in the previous articles, it was not included in the variable discussion this time. However, atropine is clinically used to treat myopia and is administered according to myopic refraction; children with higher myopia will need more frequent and higher dosages of atropine. Even when myopia occurs earlier, the treatment period will be longer. Therefore, this article currently takes the total cumulative dosage and total medication period into consideration to reflect the relative initial degree of myopia. Patients were divided into two subgroups based on a base IOP cutoff of 14 mmHg, with 619 eyes with a base IOP ≤14 mmHg and 572 eyes with a base IOP >14 mmHg. Base IOP was measured using a noncontact tension test (Xpert NCT plus, Reichert, Inc., Depew, NY, USA) and refractive error was assessed using a Canon RK5 autorefractor auto keratometer (Canon Co., Ltd., Tochigi, Japan). The visualization process for preparing the data is shown in Figure 1.

### 2.2. Variable Definition and Data Description

The study evaluates eight variables (X1–X8) that might affect the end IOP (Y), including sex (X1); age (X2); total follow-up duration (X3); the total cumulative dose of atropine (X4); previous duration (X5); the previous cumulative dose (X6) of atropine use before 1 January 2008; the recruit duration (X7); and the recruit cumulative dose (X8) of atropine use during the study period. This retrospective analysis reviewed patient records from their first visit after 1 January 2005 to their last visit by 30 December 2008, which is represented by X3. Atropine doses were calculated by multiplying the prescribed vial dose (5, 12.5, 25, or 50 mg) by the number of vials and summing these to determine the total cumulative dose (X4). Atropine use after 1 January 2005 but before 1 January 2008 is defined as “previous data”, which are also represented by X5 and X6. The recruit atropine treatment was from 1 January 2008 to the last visit on 30 December 2008, which is the period of X7 and X8 being collected. The end IOP (target Y) was recorded as the value from the last visit before the termination of data collection. The descriptive statistics in each subgroup are presented in Table 1.

### 2.3. SHapley Additive exPlanations (SHAP)

The interpretability of an ML model may be limited when it comes to explaining individual cases, as ML typically provides a macro/general perspective on the overall data structure. Moreover, the mechanisms could be too complex for clinicians for a straightforward understanding, particularly when trying to discern how specific features influence a target outcome. To address these challenges and improve interpretability, SHAP was utilized in this study. SHAP is a method designed to explain individual predicted outcomes from an ML model by extending the concept of Shapley values from cooperative game theory [19]. SHAP assigns a contribution value to each feature by comparing predictions with and without the feature. This approach considers all possible combinations of features and shows how each feature impacts the predicted outcome, either positively or negatively. Overall, SHAP provides detailed insights into how features influence predictions and has become increasingly popular in clinical studies for explaining feature effects [20,21].

### 2.4. ML Methods

To examine how features affect end IOP predictions in myopic patients treated with atropine, ML methods were applied for their effectiveness in handling complex feature interactions and common usage in clinical studies [22]. Four ML methods included least absolute shrinkage and selection operator regression (Lasso), classification and regression tree (CART), extreme gradient boosting (XGB), and random forest (RF), which are utilized in this study [23,24,25,26]. Each of the utilized ML methods in this study employs distinct strategies to enhance predictive accuracy and feature selection.

Lasso is an extended linear regression method that employs the L1 regularization technique, penalizing the absolute value of coefficients. This penalty encourages sparsity in the model by shrinking less influential coefficients toward zero, effectively selecting only the most impactful features. Lasso’s inherent feature selection mechanism makes it particularly effective in reducing overfitting and improving interpretability in high-dimensional datasets.

CART constructs a single regression tree by recursively splitting the data based on variable cutoff points that minimize the prediction error at each step. This approach results in an interpretable decision tree structure, where each split represents a condition on the input variables, ultimately predicting the outcome at the terminal nodes. Furthermore, when constructing the decision tree, the splitting process inherently prioritizes variables that contribute the most to reducing prediction errors, often leaving less relevant features being ignored into the final tree structure.

XGB takes an iterative approach to improving model performance, also known as a boosting approach. By iteratively correcting the residual errors of the previous model under the structure of boosting, XGB enhances accuracy while maintaining computational efficiency. It integrates advanced techniques such as Taylor binomial expansion to speed up convergence, regularization to prevent overfitting, and parallel processing to optimize resource utilization. When constructing XGB, it identifies features that contribute most to reducing the overall error, assigning higher importance to these features through weighted updates. Additionally, XGB includes built-in feature importance metrics, such as gain and cover, to quantify each variable’s contribution to the model’s performance.

RF is a robust ensemble method that extends the CART approach by building multiple regression trees using randomly selected subsets of the training data and variables. The final prediction from RF is obtained by averaging the predictions from all the individual trees in the forest, reducing variance and improving generalization. This ensemble technique increases robustness and minimizes the likelihood of overfitting compared to a single decision tree. In addition, RF evaluates feature importance based on how much each feature reduces the prediction error across all trees in the forest, with less significant features being ignored or less impactful among the overall RF model.

### 2.5. Modeling Scheme

**Figure 2:**
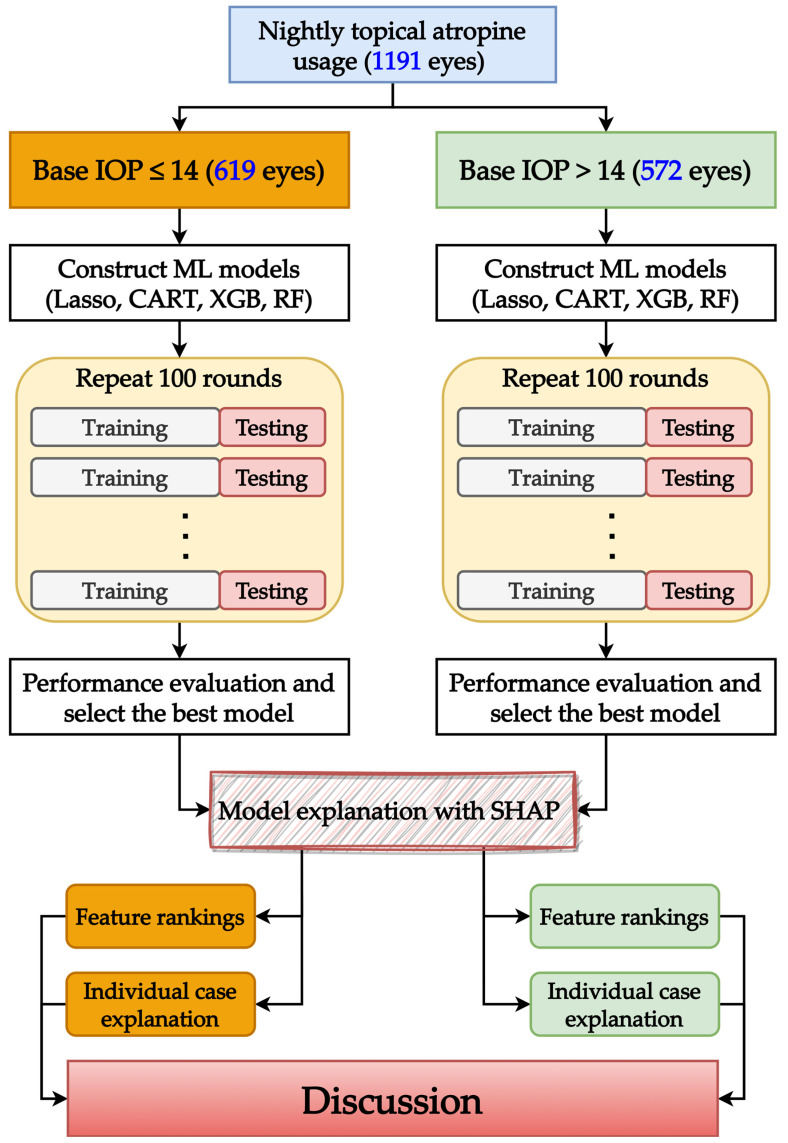
Modeling scheme.

Figure 2 illustrates the modeling scheme of this study, which consists of two parts for each base IOP subgroup. The first part involved training the model for 100 rounds to identify the best-performing model; the second part used SHAP to interpret the identified model. As shown in Figure 2, after preparing the base IOP subgroups (as in Figure 1), ML models were repeatedly built. When building models in each round, each ML method required hyper-parameters to be tuned, which is achieved through five-fold cross-validation (5f-CV). 5f-CV randomly divided the training data into five equal folds, with four used for training and one for validation, rotating until all folds were validated. The best hyper-parameter set was chosen based on average validation results, and the model was then tested. Finally, SHAP explained the model with the best testing performance by providing the overall feature rankings and individual case explanations.

The root mean squared error (RMSE), mean absolute percentage error (MAPE), symmetric mean absolute percentage error (SMAPE), relative absolute error (RAE), and root relative squared error (RRSE) were considered for performance evaluation. Additionally, the experiments in this study were implemented using Python software (version 3.8.8) and related packages.

## 3. Results

### 3.1. ML Model Results

To validate the use of ML methods, a multiple linear regression (MLR) model, a standard in clinical studies, was also developed for comparison. Table 2 presents the mean and standard deviation (SD) of 100-round model errors for both base IOP subgroups. In the base IOP ≤14 subgroup, RF outperformed MLR and all other models across all five error metrics. In the base IOP >14 subgroup, Lasso performed similarly to MLR, while all the other ML models outperformed MLR. XGB and RF had a comparable MAPE, SMAPE, and RAE, but RF showed the best RMSE (2.37) and RRSE (0.97). Given RF’s superior performance in the base IOP ≤14 subgroup and its better RMSE and RRSE in the base IOP >14 subgroup, RF was determined to be the best model for further explanation with SHAP.

### 3.2. Feature Importance from SHAP in Each Subgroup

**Figure 3:**
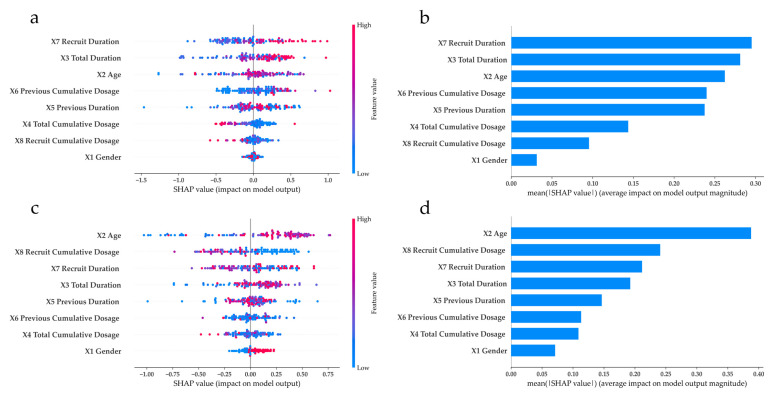
SHAP summary and feature importance plot of each base IOP subgroup. (**a**) SHAP summary plot of base IOP ≤14 subgroup. (**b**) SHAP feature importance plot of base IOP ≤14 subgroup. (**c**) SHAP summary plot of base IOP >14 subgroup. (**d**) SHAP feature importance plot of base IOP >14 subgroup.

Using SHAP to interpret the best RF model, the overall impact of each feature on end IOP in different base IOP subgroups was visualized, as shown in Figure 3. Panels (a) and (b) correspond to the base IOP ≤14 subgroup, while panels (c) and (d) correspond to the base IOP >14 subgroup. Panel (a) shows a SHAP summary plot, where each dot represents the SHAP value for a feature affecting an individual case, with an increasing or decreasing impact on end IOP. The SHAP value of zero serves as a datum point (DP). Dots to the right of DP suggest an increased impact, while those to the left suggest a decreased impact. The color bar on the right displays the actual high/low feature values of an individual case, with higher values appearing in red and lower values in blue.

The SHAP summary plot provides clinicians an overview of how high/low values of features are likely to affect the end IOP outcome for an individual case. Panel (a) shows that cases with higher values of X7 and X3 are more likely to cause increased end IOP outcomes, as indicated by the concentration of red dots to the right of DP. Moreover, X5 has a mixed influence, varying between cases. To determine the overall impact of a feature, panel (b) averages the absolute SHAP values from panel (a), helping clinicians prioritize the features to be focused. The same concept is followed for panels (c) and (d). In panel (c), it can be found that a lower value of X8 is likely to have increased impact on end IOP, while other medication-related features show mixed effects. Comparing panels (b) and (d), the features have various impacts on the end IOP in different base IOP subgroups. For example, X8 has a greater impact on end IOP in the base IOP >14 subgroup than in the base IOP ≤14 subgroup. Overall, with the aid of SHAP, helpful insights can be generated to learn more about the relationship between atropine use and end IOP.

### 3.3. Demonstrations of Individual Case Explanation with SHAP for Each Subgroup

**Figure 4:**
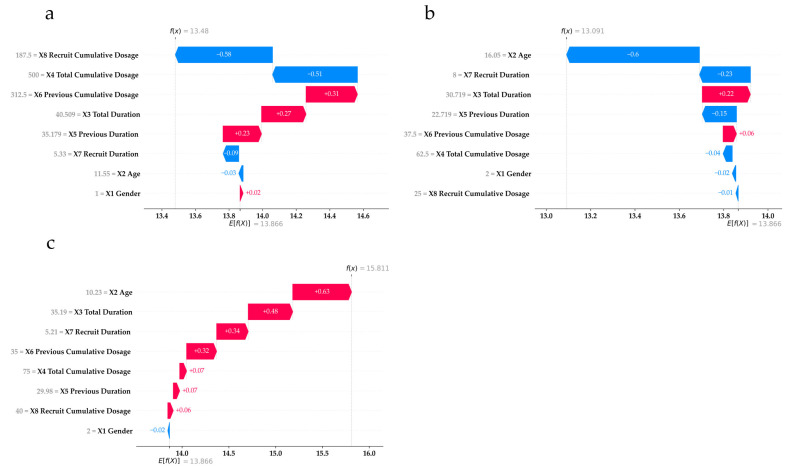
Three examples of individual case (panels (**a**–**c**)) explanations in base IOP ≤14 subgroup. $f\left(x\right)$: model prediction outcome. $E\left[f\left(x\right)\right]$: expected value.

**Figure 5:**
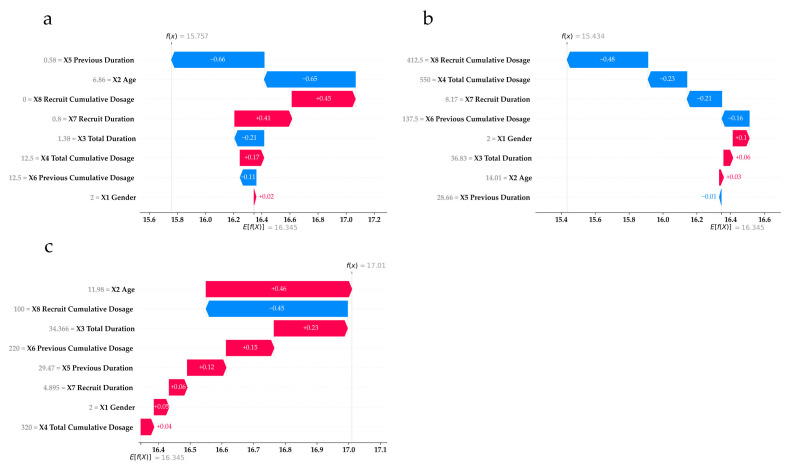
Three examples of individual case (panels (**a**–**c**)) explanations in base IOP >14 subgroup. $f\left(x\right)$: model prediction outcome. $E\left[f\left(x\right)\right]$: expected value.

Understanding how feature values affect end IOP at the individual level is vital for clinicians when planning treatment and determining medication dosages. SHAP aids this process by showing how each feature value influences end IOP outcomes in individual cases using waterfall plots (Figure 4 and Figure 5). Figure 4 presents the example cases in the base IOP ≤14 subgroup, while Figure 5 presents the example cases in the base IOP >14 subgroup.

Consider panel (a) of Figure 4 as an example for demonstrating how to interpret the waterfall plots. The horizontal axis represents the end IOP value, while the red and blue bars display the increasing and decreasing SHAP values, respectively, representing the contribution of each feature to the final model predicted outcome ($f\left(x\right)$, located at the top of the plot). The expected value ($E\left[f\left(x\right)\right]$, located at the bottom of the plot) is the average end IOP from the data used to train the model, which represents the prediction made without using any features. The final predicted outcome is obtained by adding the expected value and each feature’s SHAP value. The vertical axis lists the features and their actual values for each case. Hence, in panel (a), X8 and X4 cause the largest decrease in end IOP for this case.

SHAP helps visualize how feature influences vary between individuals. For example, in panels (e) and (f) of Figure 4, the predicted outcomes are similar (end IOP = 13), but the key features differ. For the case in panel (a), X8 and X4 contributed the most; in panel (b), the largest contributions were from X2 and X7. Moreover, panels (b) and (c) show that even when the same feature (X2) is most influential, impact can vary with different feature values. X2 causes a decreasing impact on end IOP in panel (b) but causes an increasing impact in panel (c).

The same concept applies to Figure 5 for the base IOP > 14 subgroup. For instance, panels (a) and (b) show similar prediction outcomes resulting from different feature contributions. Notably, in panel (c), X2 and X8 offset each other, with X3 being the largest increasing impact contributor. In summary, SHAP illustrates how features and their values influence end IOP outcomes from RF, providing clinicians with detailed insights to better anticipate a patient’s potential end IOP with prescribed medications.

## 4. Discussion

ML models combined with SHAP can rank risk factors and interpret model results [20,21,27]. This study is the first to use ML algorithms with SHAP to analyze confounders across two baseline IOP groups and assess each factor’s impact on predicting final IOP in children with myopia treated with atropine. While the average IOP in both groups remained within the normal range, the base IOP was slightly higher in the older group (Table 1) and the end IOP was higher in the base IOP >14 subgroup. The RF analysis model showed that age (X2) was the most important factor, followed by the cumulative dose (X8) and duration (X7) of atropine (Figure 3, panel [d]). Therefore, older children within base IOP >14 mmHg, their cumulative dosage, and duration of atropine treatment should be closely monitored [28].

The group with a base IOP ≤ 14 mmHg is slightly younger, which may contribute to the lower base IOP (Table 1). RF analysis shows that recruit duration (X7) and total duration (X3) are the most important factors influencing end IOP after atropine use (Figure 3, panel [b]). This suggests that even with a lower base IOP in younger patients, a longer history of atropine use could increase the overall treatment duration. Therefore, close monitoring of IOP is essential in long-term atropine therapy, emphasizing the importance of using low-dose atropine to minimize cumulative side effects [7,29].

In panels (b) and (d) of Figure 3, the two groups of children with myopia with different base IOPs, the confounding factors of final IOP, age (X2), and recruit duration (X7) appear among the first three main effects. When using atropine, the longer the medication is taken, the more closely changes in children’s IOP need to be monitored. Li et al. [11] conducted a follow-up for the ATOM1 study (atropine 1% vs. placebo; 1999–2003) and the ATOM2 study (atropine 0.01% vs. 0.1% vs. 0.5%; 2006–2012). They compared the 1% atropine-treated group versus the placebo group for the 20-year incidence of cataract/lens opacities, myopic macular degeneration, or parapapillary atrophy (β/γ zone). For children with myopia on long-term atropine, potential sequelae should be monitored and long-term safety tracking is necessary [11].

Our team’s previous study, “Identifying and Exploring the Impact Factors for Intraocular Pressure Prediction in Myopic Children with Atropine Control Utilizing Multivariate Adaptive Regression Splines”, demonstrated through the MARS model, showed that a baseline intraocular pressure (IOP) exceeding 14 mmHg is associated with an increased risk of elevated end IOP [18]. Building on this finding, the present study provides a more in-depth analysis and extended discussion. In the current study, the average intraocular pressure for the entire cohort changed from an initial 14.5 mmHg to a final 15.07 mmHg, with an average difference of 0.53 mmHg, consistent with our previous findings. Prior biostatistical studies, including both retrospective and prospective analyses, on children with myopia treated with atropine have consistently shown that atropine treatment does not significantly increase the risk of IOP exceeding safety thresholds associated with glaucoma development [16,17,18].

Given that the significance of baseline spherical equivalent (base SE) was less prominent in earlier studies [17,18], it was not addressed in the current discussion. However, it is well established that the incidence of glaucoma, including normotensive glaucoma, is higher in individuals with myopia [11,28]. Clinically, changes in refraction may influence both treatment regimens and their impact on final intraocular pressure. Future research should focus on a more detailed investigation of refractive changes in relation to myopic progression and their implications for treatment outcomes.

SHAP provided a dynamic RF analysis explanation module, including variables like age, sex, and the duration and dosage of atropine treatment. This study aimed to avoid the risk of a possible increase in IOP when using atropine to treat children with myopia, enabling individualized monitoring of medication safety. In the future, this module could potentially help preset clinical treatment plans based on patient characteristics, whereas the treatment effects can also be monitored as well as the adjustment of therapies if needed. Hence, the module offers clinicians a more precise information policy that would enable more customized and safer treatment. Overall, we hope the SHAP module will help physicians develop more customized and precise atropine treatment plans for myopia control. After treatment, physicians could adjust factors to better meet individual needs, balancing efficacy and safety.

Previously, we used biometric and MARS methods to study the impact of atropine on IOP in children with myopia, finding that the long-term use of atropine alone does not significantly increase IOP [16,17,18]. However, in recent years, low concentrations of atropine (0.01%) have been combined with corneal reshaping to treat myopia [30]. This combined treatment method has a case report stating that there is a risk of increased IOP [31]. Studies suggest that IOP measurements after removing OK lenses may be falsely lowered due to corneal flattening [32,33]. Therefore, if clinicians encounter atropine and OK lenses combined to treat children with myopia in the future, they must pay more attention to the prediction and tracking of IOP. This article focuses on children with myopia who use atropine alone and reveals the treatment duration and dosage, as well as the patient’s personal conditions, such as age, as important variables for the final end IOP. In the future, if myopic treatment becomes more complicated, such as a combination of atropine and OK lenses, more variables can be added to the SHAP module to predict and monitor IOP [5,6,31].

Some limitations of this study need to be addressed. This study’s data were hospital-based, with only 1191 eyes analyzed. The predictive accuracy of the SHAP module could further be verified with larger datasets if available. Moreover, atropine is commonly used for treating myopia in children due to the increasing incidence of myopia, which can persist into adolescence. However, the study’s treatment duration was only 4 years. Future research should investigate the model’s predictive value for IOP over a longer treatment period.

## 5. Conclusions

This study is the first, to our knowledge, to use the SHAP module to analyze children with myopia treated with topical atropine. SHAP created a personalized graphic illustrating how various factors such as age, sex, and both the previous and cumulative duration and dosage of atropine affect end IOP. In different base IOP subgroups, age (X2) was the most important factor in the base IOP >14 subgroup, whereas recruit duration (X7) was the one in the base IOP ≤14 subgroup. This approach offers clinicians individualized insights to enhance medication safety and patient management.

## Figures and Tables

**Table 1:** Descriptive statistics of data subgroups.

Variable	Data Subgroups	
	Base IOP ≤ 14	Base IOP> 14
	**N (%)**	
X1: Sex		
1: Male	329 (53%)	310 (54%)
2: Female	290 (47%)	262 (46%)
	**Mean (SD)**	
X2: Age	10.28 (2.46)	10.96 (2.50)
X3: Total Duration (Months)	19.10 (12.25)	20.31 (12.33)
X4: Total Cumulative Dosage (mg)	116.47 (135.37)	117.62 (137.40)
X5: Previous Duration (Months)	13.54 (12.46)	14.61 (12.37)
X6: Previous Cumulative Dosage (mg)	78.17 (107.27)	77.30 (112.09)
X7: Recruit Duration (Months)	5.53 (3.78)	5.70 (3.72)
X8: Recruit Cumulative Dosage (mg)	38.30 (59.80)	40.32 (63.54)
Y: End IOP (mmHg)	13.83 (2.49)	16.41 (2.44)

**Table 2:** Results of ML models with different subgroup data.

Subgroup	Model	RMSE	MAPE%	SMAPE%	RAE	RRSE
		Mean (SD)				
Base IOP ≤14	MLR	2.45 (0.16)	14.18 (1.00)	13.64 (0.85)	1.00 (0.02)	1.00 (0.02)
	Lasso	2.44 (0.16)	14.12 (1.00)	13.59 (0.86)	0.99 (0.02)	1.00 (0.01)
	CART	2.47 (0.16)	14.29 (0.98)	13.76 (0.84)	1.01 (0.03)	1.01 (0.03)
	XGB	2.36 (0.15)	13.36 (0.94)	13.02 (0.81)	0.96 (0.05)	0.96 (0.04)
	RF	2.30 (0.15)	13.14 (0.95)	12.74 (0.81)	0.93 (0.05)	0.94 (0.04)
Base IOP >14	MLR	2.45 (0.20)	12.00 (0.92)	11.69 (0.86)	1.01 (0.02)	1.01 (0.02)
	Lasso	2.45 (0.20)	12.02 (0.91)	11.70 (0.84)	1.01 (0.02)	1.01 (0.01)
	CART	2.43 (0.19)	11.92 (0.91)	11.60 (0.85)	1.00 (0.03)	1.00 (0.03)
	XGB	2.39 (0.19)	11.42 (0.79)	11.27 (0.77)	0.97 (0.04)	0.98 (0.04)
	RF	2.37 (0.19)	11.58 (0.89)	11.30 (0.82)	0.97 (0.05)	0.97 (0.04)

## Data Availability

The datasets generated and/or analyzed during the current study are not publicly available due to privacy/ethical restrictions but are available from the corresponding author upon reasonable request.
